# Marked Improvement of Palmoplantar Pustulosis After the Reintroduction of Apremilast Following Initiation of Dental Infection Control and Periodontal Therapy

**DOI:** 10.7759/cureus.115142

**Published:** 2026-08-25

**Authors:** Masamoto Murakami, Shuhei Yamamoto, Yuka Nojiri, Kento Kojima, Takahiko Morotomi

**Affiliations:** 1 Department of Dermatology, Nagoya City University Midori Municipal Hospital, Nagoya, JPN; 2 Department of Dermatology, Tokyo Medical University, Tokyo, JPN; 3 Department of Anatomy, Histochemistry and Cell Biology, University of Miyazaki, Miyazaki, JPN; 4 Department of Dermatology, Nagoya City University, Nagoya, JPN; 5 Department of Periodontology, Aichi Gakuin University, Nagoya, JPN; 6 Department of Endodontics, Aichi Gakuin University, Nagoya, JPN

**Keywords:** almoplantar pustulosis, apremilast, focal infection, odontogenic infection, periodontal therapy

## Abstract

Palmoplantar pustulosis (PPP) is a chronic inflammatory disorder characterized by recurrent sterile pustules, erythema, scaling, and hyperkeratosis on the palms and soles. Oral infectious foci and periodontal inflammation have been implicated as aggravating factors, particularly in Japanese patients. We report the case of a 60-year-old woman with longstanding PPP and a previous course of apremilast that she had discontinued because of perceived insufficient benefit. At presentation, the Palmoplantar Pustulosis Area and Severity Index (PPPASI) score was 37.8. Dental evaluation identified multiple periapical lesions, an endodontic-periodontal lesion, and generalized periodontitis, Stage III, Grade C. During initial periodontal therapy, including oral hygiene instruction and supragingival scaling, the periodontal inflamed surface area decreased from 1,894.5 to 558.8 mm², and the PPPASI score decreased to 20.5 approximately six months after the initiation of dental treatment, corresponding to a 45.8% improvement that did not meet the PPPASI-50 threshold. At that time, apremilast had not yet been reintroduced, and definitive endodontic treatment had not been completed; root canal treatment of tooth 47 was initiated at approximately month six and completed at month eight. Because residual PPP persisted and subsequent improvement slowed, apremilast was reintroduced approximately two months after the month six assessment at 30 mg/day. Two days later, transient acute urticaria occurred, and apremilast was temporarily interrupted. The eruption resolved by the following day without additional treatment or systemic hypersensitivity features. Because of the patient’s previous history of urticaria and the absence of recurrence after rechallenge, the episode was considered unlikely to represent apremilast hypersensitivity. Apremilast was restarted at 30 mg/day, and approximately 2.5 months after the reintroduction, the PPPASI score had decreased to 6.1, corresponding to an 83.9% improvement from baseline and achievement of PPPASI-75. This single case provides a hypothesis-generating observation that reduction in periodontal inflammatory burden was temporally associated with partial improvement before apremilast reintroduction, after which further marked improvement was observed. Evaluation and management of oral infectious foci and periodontal inflammation may therefore be considered an adjunctive strategy in selected patients with PPP who have a suboptimal response to systemic therapy, particularly when clinically or radiographically evident oral disease is present.

## Introduction

Palmoplantar pustulosis (PPP) is a chronic relapsing inflammatory disorder characterized by recurrent sterile pustules, erythema, scaling, and hyperkeratosis on the palms and soles. PPP is more common in women, often develops in middle age or later, and is strongly associated with smoking. In Japan, focal infections are recognized as clinically important triggers or aggravators in a substantial proportion of patients [1].

Oral disease relevant to PPP may include both periapical/endodontic infectious foci and periodontitis, but these entities should not be used interchangeably. Periapical disease refers to pathology around the root apex, often associated with endodontic infection, whereas periodontitis is a chronic inflammatory disease of the tooth-supporting tissues. Kouno et al. reported clinical improvement in 44 of 70 patients with PPP (63%) who underwent dental infection control [2]. Yamashita et al. reported improvements in PPP, along with decreases in systemic inflammatory markers and periodontal inflammatory burden, during periodontal treatment [3]. Periodontitis is initiated by a dysbiotic biofilm and sustained by a dysregulated host immune response. Persistent periodontal inflammation may contribute to systemic immune activation through bacterial products and inflammatory mediators, including interleukin (IL)-1β, IL-6, tumor necrosis factor-alpha (TNF-α), and IL-17. Several of these inflammatory pathways overlap with those implicated in PPP; however, a direct causal relationship between periodontitis and PPP has not been established [1,4].

Apremilast is an oral phosphodiesterase 4 inhibitor that increases intracellular cyclic adenosine monophosphate and modulates a network of pro- and anti-inflammatory mediators rather than targeting a single cytokine pathway [5]. In Japanese patients with PPP, phase 2 and phase 3 randomized trials demonstrated clinical efficacy of apremilast, including improvement in Palmoplantar Pustulosis Area and Severity Index (PPPASI)-based outcomes [6-8].

However, reports describing apremilast reintroduction in the setting of concurrent periodontal and endodontic management remain limited. The present case is clinically informative because the reduction in periodontal inflammatory burden and partial improvement in PPP preceded completion of definitive endodontic treatment, and the reintroduction of apremilast was subsequently associated with additional marked improvement.

## Case presentation

A 60-year-old woman had developed scaling, vesicles, and pustules on the soles approximately 15 years before presentation. She had smoked 20 cigarettes per day for more than 30 years and had stopped smoking approximately six months before her first visit to our hospital. Previous treatments at a local dermatology clinic included biotin, phototherapy, and removal of dental metal restorations. She had no articular symptoms, and her tonsils had not been evaluated as a potential source of focal infection.

At a previous university hospital, apremilast had been prescribed for approximately 18 months. It was initiated at 60 mg/day and was later reduced to 30 mg/day. Approximately two months before presentation to our hospital, the patient stopped taking apremilast on her own because she perceived insufficient benefit. Objective PPPASI scores and standardized clinical photographs from the previous treatment period were unavailable, and she had experienced no urticaria or other hypersensitivity reactions during that initial course.

At presentation to our department (month zero), pustules, erythema, scaling, and hyperkeratosis were present on both palms and soles, with more severe disease on the soles (Figure 1).

**Figure 1:**
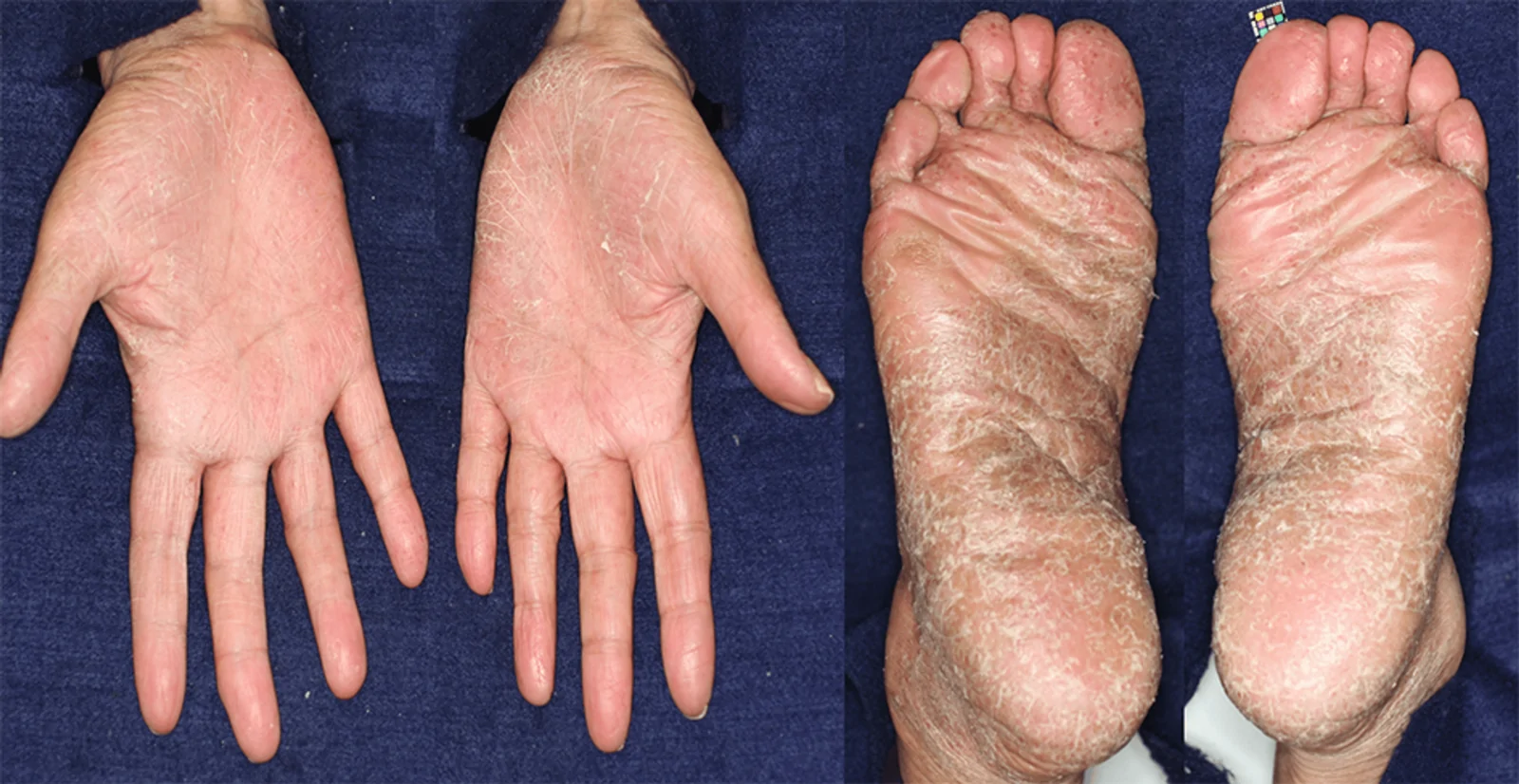
Clinical findings at the initial visit. Pustules, erythema, scaling, and hyperkeratosis were seen on both palms and soles. The Palmoplantar Pustulosis Area and Severity Index (PPPASI) score was 37.8.

To confirm the diagnosis, a skin biopsy was obtained from the left sole. Histopathological examination showed intraepidermal pustulovesicles and spongiform pustules of Kogoj, without prominent spongiosis suggestive of eczema (Figure 2).

**Figure 2:**
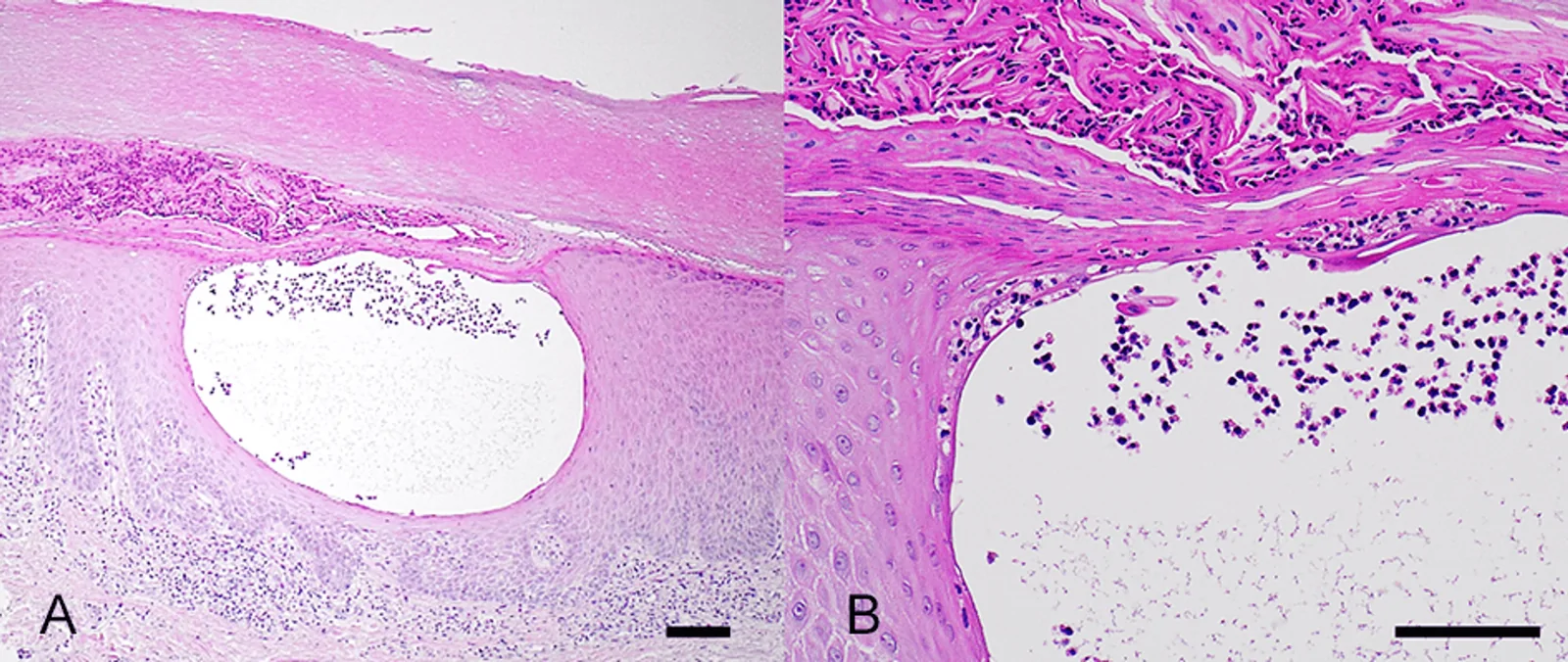
Histopathological findings at the initial visit. A biopsy specimen from the left sole showed an intraepidermal pustulovesicle and spongiform pustules of Kogoj without prominent eczematous spongiosis. A: low-power field (×100). B: high-power field (×400). Hematoxylin-eosin staining. Scale bars: 100 μm.

Disease severity was assessed using PPPASI, calculated according to the method originally described by Bhushan et al. [6]. Only numerical PPPASI results are reported; the instrument, scoring form, instructions, calculation table, and translated versions are not reproduced. The total PPPASI score was 37.8, with a palmar subscore of 9.0 and a plantar subscore of 28.8. Metal patch testing was negative. During the subsequent dental treatment interval, topical difluprednate and excimer light therapy were maintained without major changes.

Because oral infectious and inflammatory disease was considered a potential aggravating factor, dental evaluation was requested on day 21. At approximately month one, a panoramic radiograph was obtained during the initial endodontic evaluation, and full-mouth periapical radiographs were obtained at the subsequent periodontal evaluation (Figure 3).

**Figure 3:**
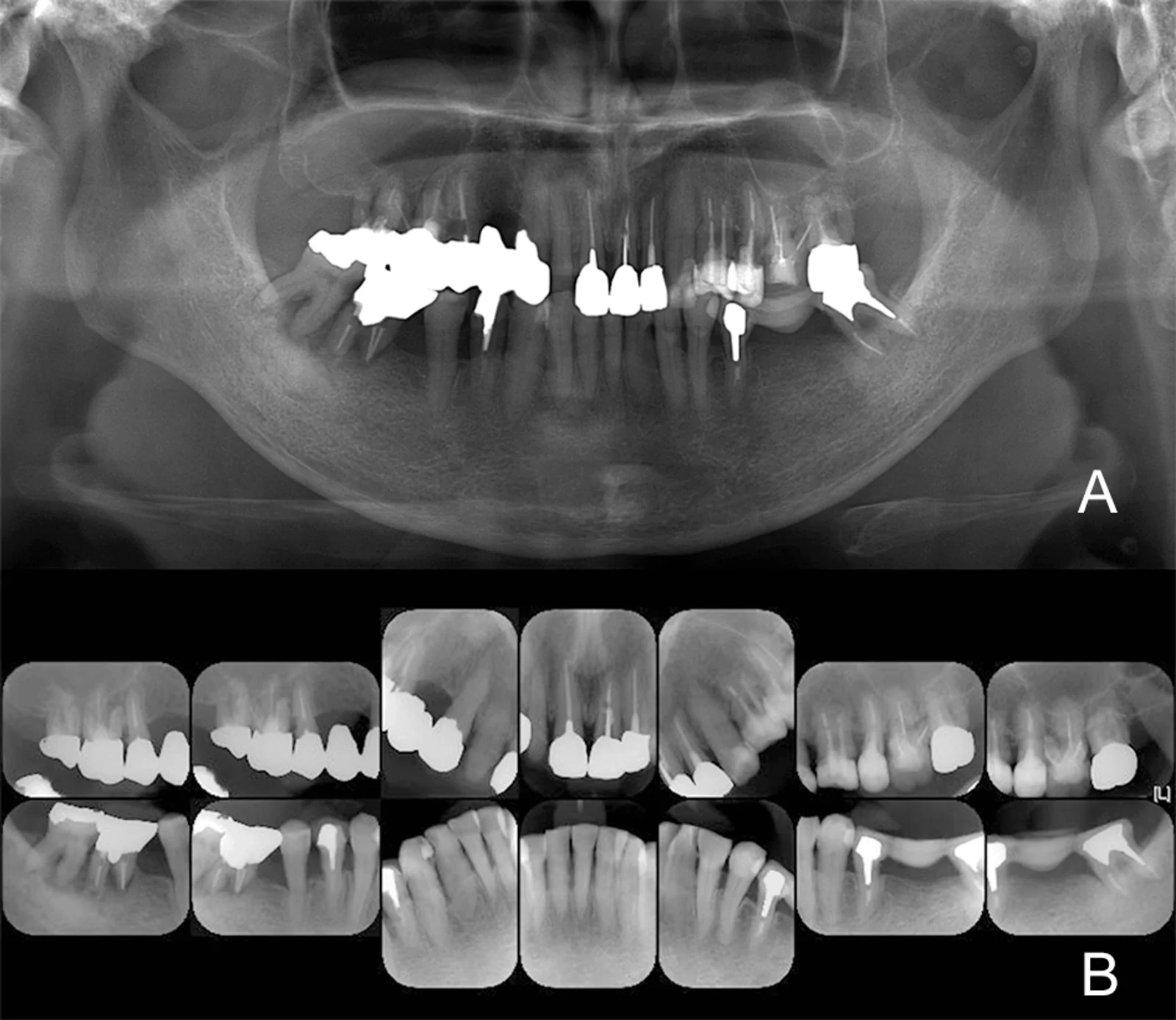
Radiographic findings at the initial dental evaluation. A: Panoramic radiograph obtained during the initial endodontic evaluation. B: Full-mouth periapical radiographs obtained at the subsequent periodontal evaluation. Periapical radiolucencies were identified in teeth 16, 17, 26, 27, 46, and 47, consistent with chronic apical periodontitis. Tooth 17 also had a deep periodontal pocket and was diagnosed as an endodontic-periodontal lesion.

Periapical radiolucencies were identified in teeth 16, 17, 26, 27, 46, and 47 (FDI notation), consistent with chronic apical periodontitis. Tooth 17 also had a deep periodontal pocket and was diagnosed as an endodontic-periodontal lesion.

Periodontal examination was performed at six sites per tooth, for a total of 156 sites. The mean probing pocket depth (PPD) was 4.2 mm; 37.2% of sites (58 sites) had a PPD of 4-5 mm and 15.4% (24 sites) had a PPD of 6 mm or greater. Bleeding on probing (BOP) was present at 76.2% of sites (119 sites). Periodontal inflamed surface area (PISA), which estimates the inflamed periodontal epithelial surface area and serves as a surrogate measure of whole-mouth periodontal inflammatory burden, was 1,894.5 mm² [9]. The O'Leary Plaque Control Record (PCR) was 48.1% [10], indicating marked plaque accumulation, particularly in interdental areas.

According to the 2017 World Workshop staging and grading framework, the periodontal diagnosis was generalized periodontitis, Stage III, Grade C [11]. Supporting findings included clinical attachment loss of at least 5 mm, radiographic bone loss extending to the middle third of the root, no more than four teeth lost because of periodontitis, vertical bone defects of at least 3 mm, Class III furcation involvement, and a bone-loss/age ratio greater than 1.0. Although some teeth showed mobility of degree 2 or greater, the occlusal relationship remained stable and complex occlusal rehabilitation was not required; therefore, the disease was classified as Stage III rather than Stage IV.

Initial periodontal therapy began at approximately month three and included oral hygiene instruction and supragingival scaling. Oral hygiene instruction was provided in accordance with the method described by Mitani et al. [12]. After this initial therapy, the PCR improved to 19.2%, mean PPD to 3.2 mm, the proportion of sites with PPD of 4-5 mm to 25.6% (40 sites), the proportion with PPD of 6 mm or greater to 5.8% (9 sites), BOP to 28.2% (44 sites), and PISA to 558.8 mm², representing a 70.5% reduction (Figure 4; Table 1). PPD and BOP provide site-specific information regarding periodontal pocketing and bleeding. In contrast, PISA integrates bleeding pocket measurements across the dentition into a single estimate of whole-mouth periodontal inflammatory burden. PISA was therefore useful for longitudinal assessment of the overall periodontal inflammatory burden in this patient.

**Figure 4:**
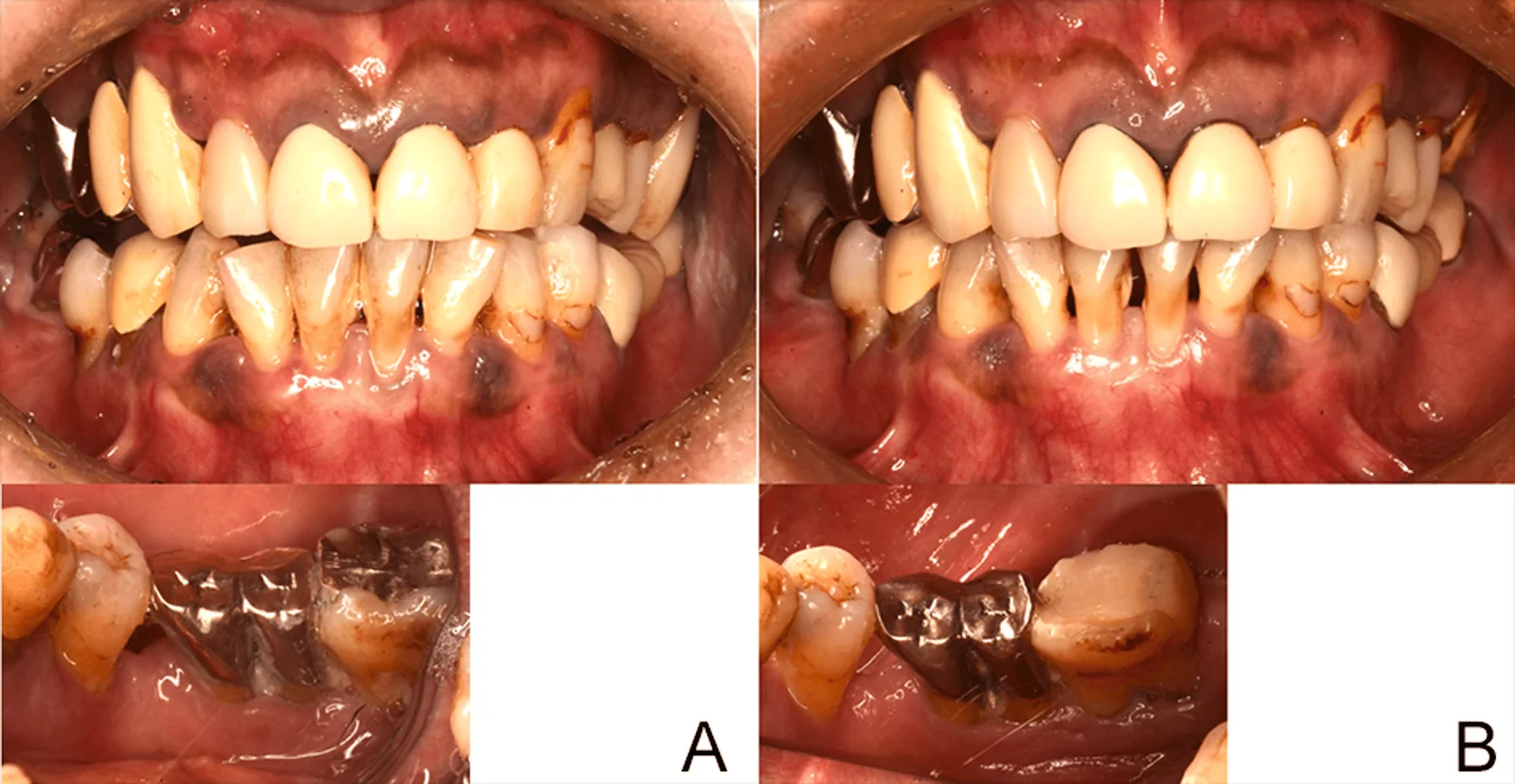
Changes in gingival inflammation and periodontal parameters during periodontal therapy. Representative intraoral photographs at the initial periodontal evaluation (A) and during initial periodontal therapy (B). After oral hygiene instruction and scaling, gingival swelling and inflammatory changes were reduced. O'Leary Plaque Control Record (PCR) improved from 48.1% to 19.2%, bleeding on probing (BOP) from 76.2% to 28.2%, and periodontal inflamed surface area (PISA) from 1894.5 mm² to 558.8 mm².

**Table 1:** Periodontal findings and response to periodontal therapy. PCR = O'Leary Plaque Control Record; PPD = probing pocket depth; BOP = bleeding on probing; PISA = periodontal inflamed surface area

Parameter	Initial dental evaluation	After initial periodontal therapy	Change
PCR	48.1%	19.2%	Improved plaque control
Mean PPD	4.2 mm	3.2 mm	Reduced pocket depth
PPD 4–5 mm	37.2% (58 sites)	25.6% (40 sites)	Reduced
PPD ≥6 mm	15.4% (24 sites)	5.8% (9 sites)	Reduced
BOP	76.2% (119 sites)	28.2% (44 sites)	Reduced gingival inflammation
PISA	1894.5 mm²	558.8 mm²	70.5% reduction

At the initial dental assessment, tooth 46 was considered to have a poor prognosis and extraction was considered, while root resection of tooth 47 was also considered. After improvement during the initial periodontal therapy, early extraction was no longer deemed necessary, and a tooth-preserving approach was adopted. Root canal treatment of tooth 47 was initiated at approximately month six and completed at month eight. Treatment of tooth 27 was initiated at month 10, and treatment of tooth 26 at month 11. Teeth 46 and 17 had not yet undergone endodontic treatment, and tooth 16 had undergone neither extraction nor root canal treatment at the latest assessment. Scaling and root planing began at month 10 and remained incomplete; active periodontal therapy was therefore ongoing, and the patient had not yet entered the supportive periodontal therapy phase.

At approximately month six, during the initial periodontal therapy and before apremilast was reintroduced, the PPPASI score decreased from 37.8 to 20.5, with a palmar subscore of 4.0 and a plantar subscore of 16.5 (Figure 5; Table 2). This represented a 45.8% improvement from baseline but did not meet the PPPASI-50 threshold. The clinical photograph shown in Figure 5B was obtained at this month 6 assessment. At that time, initial periodontal therapy with oral hygiene instruction and supragingival scaling had been performed, but definitive endodontic treatment had not been completed. Root canal treatment of tooth 47 was initiated at approximately the same stage of the dental course. No major changes in topical treatment or excimer-light therapy occurred during this interval, and no major intercurrent illness or lifestyle change other than the earlier smoking cessation was documented.

**Figure 5:**
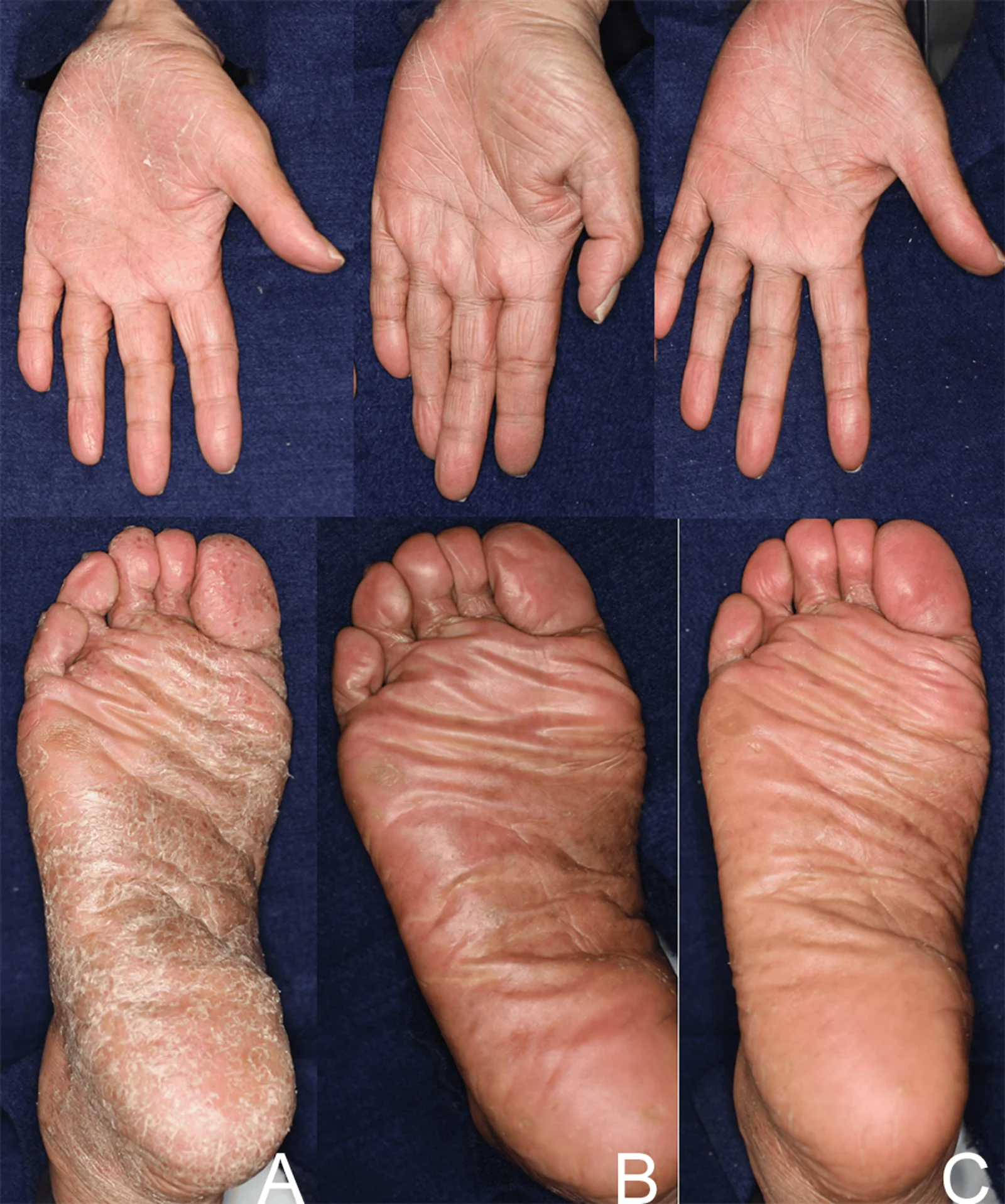
Sequential changes in palmoplantar pustulosis during treatment. Clinical images of the palms and soles at month zero (A), approximately month six during initial periodontal therapy and before apremilast reintroduction (B), and approximately 2.5 months after apremilast reintroduction (C). Palmoplantar Pustulosis Area and Severity Index (PPPASI) scores were 37.8, 20.5, and 6.1, respectively. The month six image documents the partial improvement achieved before apremilast was restarted; because further improvement subsequently slowed, apremilast was reintroduced approximately two months later.

**Table 2:** Clinical course and PPPASI response. PPPASI = Palmoplantar Pustulosis Area and Severity Index

Time point	Clinical context	Total PPPASI	Palms	Soles	Improvement from baseline
Month 0	Before dental treatment	37.8	9.0	28.8	—
~Month 6	During initial periodontal therapy; before apremilast reintroduction; definitive endodontic treatment not completed	20.5	4.0	16.5	45.8%
~Month 11	Approximately 2.5 months after apremilast reintroduction	6.1	1.6	4.5	83.9% (PPPASI-75)

After the month six assessment, residual PPP persisted, and further improvement was limited over the following approximately two months. Apremilast was therefore reintroduced at approximately month nine at 30 mg/day without titration as an add-on to the ongoing dental and dermatologic management. Two days after reintroduction, multiple small clustered wheals developed on the anterior chest, extensor upper arms, back, and dorsum of the feet. There was no pruritus, mucosal involvement, angioedema, respiratory compromise, hypotension, fever, or other evidence of anaphylaxis. Apremilast was temporarily discontinued. The urticaria resolved by the following day without additional treatment. Because the patient had a previous history of urticaria, the eruption resolved rapidly, and systemic hypersensitivity features were absent, the episode was judged unlikely to represent apremilast hypersensitivity. No allergy consultation was obtained. Apremilast was restarted several days later at 30 mg once daily. No recurrence of urticaria or other hypersensitivity reaction occurred.

Approximately 2.5 months after apremilast reintroduction (month 11), the PPPASI score decreased from 20.5 to 6.1. The palmar subscore was 1.6, and the plantar subscore was 4.5 (Figure 5C; Table 3). This represented a 70.2% improvement from the pre-reintroduction month six score and an 83.9% improvement from baseline, meeting the PPPASI-75 criterion. The clinical photograph shown in Figure 5C was obtained at this final assessment. The Dermatology Life Quality Index and other formal patient-reported outcome measures were not obtained. The patient reported that this was the first time since the onset of her disease that she had achieved a treatment result she found satisfactory. The relative clinical timeline is summarized in Table 3.

**Table 3:** Relative clinical timeline. PPPASI = Palmoplantar Pustulosis Area and Severity Index; PPP = palmoplantar pustulosis; SPT = supportive periodontal therapy

Relative time	Dermatologic course	Dental/periodontal course
Before presentation	Previous apremilast course of approximately 18 months; initially 60 mg/day, later reduced to 30 mg/day	—
~Month -2	Patient stopped apremilast on her own because of perceived insufficient benefit	—
Month 0	First visit; PPPASI 37.8 (palms 9.0, soles 28.8)	—
Day 21 to Month 1	Dental referral	Panoramic radiography and periodontal evaluation; full-mouth periapical radiographs obtained subsequently
Month 3	Stable topical difluprednate and excimer-light therapy	Oral hygiene instruction and supragingival scaling
~Month 6	Figure 5B obtained; PPPASI 20.5 (45.8% improvement; PPPASI-50 not achieved); apremilast not yet reintroduced	Initial periodontal therapy ongoing; root canal treatment of tooth 47 initiated; definitive endodontic treatment not yet completed
Month 8	Residual PPP persisted; further improvement after the month six assessment was limited	Root canal treatment of tooth 47 completed
~Month 9	Apremilast reintroduced at 30 mg/day because residual PPP persisted and improvement had slowed; transient urticaria two days later; temporary interruption; then restarted without recurrence	Active periodontal therapy continued
Month 10	—	Treatment of tooth 27 and scaling/root planing initiated
Month 11	Figure 5C obtained; PPPASI 6.1 approximately 2.5 months after apremilast reintroduction	Treatment of tooth 26 initiated; active periodontal therapy ongoing; SPT not yet started

## Discussion

This case provides a hypothesis-generating observation regarding the relationship among periodontal inflammatory burden, oral infectious foci, and systemic therapy in PPP. Initial dental evaluation revealed multiple periapical lesions and generalized periodontitis, Stage III, Grade C. During initial periodontal therapy, PISA decreased by 70.5%, and PPPASI decreased by approximately 45.8% from 37.8 to 20.5 by month six. Importantly, this partial skin improvement occurred before apremilast reintroduction and before definitive endodontic treatment had been completed. Further spontaneous improvement was limited over the next approximately two months, prompting reintroduction of apremilast at approximately month nine for residual disease. Approximately 2.5 months later, the PPPASI score had decreased further to 6.1.

The distinction between periapical/endodontic disease and periodontitis is important. Both can contribute to the broader oral infectious and inflammatory burden, but they represent different pathological processes. Previous studies have reported improvements in PPP after dental infection control and periodontal treatment [2,3]. In the present patient, however, the timing suggests that the early reduction in PPP activity was more closely aligned with improvement in periodontal inflammatory burden than with completion of endodontic therapy. This temporal relationship should not be interpreted as proof of causality.

Periodontitis is a dysbiotic inflammatory disease in which persistent microbial challenge and a dysregulated host response can promote local tissue destruction and systemic immune activation [4]. Inflammatory mediators involved in periodontal disease include IL-1β, IL-6, TNF-α, and IL-17-related pathways, some of which are also relevant to PPP pathobiology [1,4]. Nevertheless, the present case cannot determine whether periodontal inflammation directly amplified PPP, whether both conditions improved independently, or whether another factor influenced both. PISA was particularly useful because PPD and BOP are site-specific measures, whereas PISA integrates bleeding pocket measurements into an estimate of the whole-mouth inflamed periodontal epithelial surface area [9].

Confounding factors must be considered. The patient had stopped smoking approximately six months before presentation. Although severe PPP remained present at baseline, a delayed beneficial effect of smoking cessation cannot be excluded. Topical difluprednate and excimer-light therapy were also used and remained essentially unchanged during the interval in which PPPASI decreased from 37.8 to 20.5. Natural fluctuations in PPP are another possible explanation. Therefore, the 45.8% improvement during periodontal therapy should be interpreted as a temporal association rather than a treatment effect established by this case.

Apremilast inhibits PDE4, increases intracellular cyclic adenosine monophosphate, and shifts inflammatory signaling toward a less pro-inflammatory profile, including modulation of TNF-α, IL-23, and anti-inflammatory mediators such as IL-10 [5]. Randomized trials in Japanese patients support its efficacy in PPP [7,8]. In this patient, the earlier apremilast course was perceived as insufficiently beneficial, although objective PPPASI data from that period were unavailable. After partial disease improvement during periodontal therapy, apremilast reintroduction was associated with an additional 70.2% reduction in PPPASI from 20.5 to 6.1. This raises the possibility that a reduced oral inflammatory burden may have contributed to improved overall disease control, allowing reconsideration of systemic therapy. Still, it does not establish that periodontal treatment restored pharmacologic responsiveness.

The transient urticarial episode after apremilast reintroduction deserves specific consideration. It occurred two days after restarting the drug, but there was no pruritus, mucosal involvement, angioedema, respiratory compromise, hypotension, fever, or anaphylaxis. The eruption resolved by the following day without additional treatment. Because the patient had a previous history of urticaria and subsequently tolerated rechallenge at the same 30 mg/day dose without recurrence, the treating dermatologist considered a causal relationship with apremilast unlikely. This individualized rechallenge should not be interpreted as a general recommendation for patients with suspected drug hypersensitivity.

This report has several limitations. First, it describes a single patient treated in Japan, limiting generalizability to other populations and healthcare settings. Second, objective PPPASI measurements and standardized photographs were not available from the initial apremilast treatment at the previous hospital. Third, formal patient-reported outcomes such as the Dermatology Life Quality Index were not collected. Fourth, smoking cessation, concomitant topical treatment, excimer-light therapy, the natural relapsing course of PPP, and other unmeasured factors may have contributed to the observed improvement. Fifth, active periodontal and endodontic treatment was still ongoing at the latest assessment, and supportive periodontal therapy had not yet begun.

Future prospective studies should longitudinally evaluate PPP severity together with PISA, other periodontal measures, inflammatory biomarkers, and, where feasible, oral microbiome profiles. Controlled studies are needed to determine whether reduction of periodontal inflammatory burden modifies clinical responsiveness to apremilast or other systemic therapies.

## Conclusions

In this patient with PPP, a marked reduction in periodontal inflammatory burden during initial periodontal therapy was temporally associated with partial improvement in skin disease, while definitive endodontic treatment remained incomplete. Reintroduction of apremilast for residual PPP was subsequently associated with further improvement in PPPASI 6.1 and achievement of PPPASI-75. This single case does not establish causality or a treatment-enhancing interaction. Evaluation and management of oral infectious foci and periodontal inflammation may nevertheless represent an important adjunctive strategy in selected patients with PPP who have a suboptimal response to systemic therapy, particularly when clinically or radiographically evident oral disease is present. Larger prospective studies are required to determine whether reduction of oral inflammatory burden alters responsiveness to systemic treatment.
